# New Elements of Operative Surgery

**Published:** 1846-04

**Authors:** 


### PART III.—BIBLIOGRAPHICAL NOTICES.

ARTICLE V.

*New Elements of Operative Surgery.* By ALF. A. L. M. VELPEAU, Surgeon of the Hospital of La Charité, &c., &c. Carefully revised, entirely remodelled, and augmented with a treatise on Minor Surgery, illustrated by over 300 engravings incorporated with the text, accompanied with an Atlas in quarto of twenty-two plates, representing the principle operative processes, surgical instruments, &c. First American from the last Paris edition. Translated by P. S. Townsend, M.D., late physician to the Seamen's Retreat, Staten Island, New York. Augmented by the addition of several hundred pages of entirely new matter, comprising all the latest improvements and discoveries in surgery, in America and Europe, up to the present time. Under the supervision of Valentine Mott, M.D., Professor of the Operations of Surgery with Surgical and Pathological Anatomy, in the University of New York, &c., &c. In three volumes Vol. II. J. & H. G. Langley. 1846. pp. 992. (From the Publishers.)

It is necessary to add but little to the title page to give the reader all the information necessary, at this moment, in relation to this work. The part by Velpeau is, or should be, already familiar to surgeons every where, but this, voluminous as it is, is nearly equalled in size by the additions of the translator under the direction of Dr. Mott. We have not yet had time for a full examination, but shall attempt, in our next No., to present to our readers a review of the more interesting additions which have been made to the original work.

In the meantime, it is scarcely necessary to recommend the work of Velpeau & Mott to all Surgeons. The third volume and atlas are announced for publication during the approaching Autumn. The price of the whole will be but $10.

